# Comparison of the diagnostic efficiency for local recurrence of rectal cancer using CT, MRI, PET and PET-CT: A systematic review protocol

**DOI:** 10.1097/MD.0000000000012900

**Published:** 2018-11-30

**Authors:** Hongsheng Shao, Xueni Ma, Ya Gao, Jiancheng Wang, Jiarui Wu, Bo Wang, Jipin Li, Jinhui Tian

**Affiliations:** a Radiology Department, Rehabilitation Center Hospital of Gansu Province; b The Second Clinical Medical College of Lanzhou University; c Evidence-Based Medicine Center, School of Basic Medical Sciences, Lanzhou University; d Gansu Provincial Hospital, Lanzhou; e Department of Clinical Pharmacology of Traditional Chinese Medicine, School of Chinese Materia Medica, Beijing University of Chinese Medicine, Beijing; f Department of Nursing, Rehabilitation Center Hospital of Gansu Province, Lanzhou, China.

**Keywords:** computed tomography, local recurrence, magnetic resonance imaging, positron emission tomography-computed tomography, positron-emission tomography, rectal cancer/neoplasm

## Abstract

**Background:**

The risk of local recurrence (LR) continues to threat patients with rectal cancer after surgery or chemoradiotherapy. The main reason is that there is frequently extensive scarring and reactive changes after radiotherapy and resection. Thus, the diagnosis of LR can be challenging. There are different imaging modalities that have been used in the follow-up of rectal cancer, including computed tomography (CT), magnetic resonance imaging (MRI), positron emission tomography (PET), and positron emission tomography-computed tomography (PET-CT) in clinical practice.

**Methods:**

We will systematically search PubMed, EMBASE, the Cochrane Library, and Chinese Biomedical Literature Database for diagnostic trials using CT, MRI, PET, and PET-CT to detect LR of rectal cancer in April, 2018. Two review authors will independently screen titles and abstracts for relevance, assess full texts for inclusion, and carry out data extraction and methodological quality assessment using the QUADAS-2 tool. We will use bivariate meta-analysis to estimate summary sensitivity, specificity, positive likelihood ratio, negative likelihood ratio, and diagnostic odds ratio of CT, MRI, PET, and PET-CT, as well as different sequences of MRI. For each index test, estimates of sensitivity and specificity from each study will be plotted in summary receive operating curve space and forest plots will be constructed for visual examination of variation in test accuracy. We will perform meta-analyses using the hierarchical summary receiver-operating characteristic model to produce summary estimates of sensitivity and specificity. Then, head-to-head and indirect comparison meta-analyses will be carried out.

**Discussion:**

This review will help determine the diagnostic accuracy of CT, MRI, PET, and PET-CT for the diagnosis of patients with LR of rectal cancer.

**Ethics and dissemination:**

Ethics approval and patient consent are not required, as this study is a systematic review.

**PROSPERO registration number:**

CRD42018104918.

## Abstract 2

Supplemental Digital Content is available in the text

## 1 Introduction

### 1.1 Target condition being diagnosed

Colorectal cancer is the third most common cancer worldwide with an estimated 1.2 million new cases per year, ultimately responsible for 8% of all cancer deaths.^[1]^ Approximately one-third of these tumors are rectal cancers. Surgery, radiotherapy and chemotherapy are regarded as the cornerstones of therapy for patients with rectal cancer.^[2]^ With the exception of very early tumors that can be managed by local excision alone, the mainstay of therapy for rectal cancer is radical surgery. Total mesorectal excision has emerged as the surgical technique that can substantially reduce local recurrences (LRs). However, the risk of distant and LRs continues to threaten patients with rectal cancer.^[3]^ Various studies have reported that LR is the most common complication in patients undergoing surgical treatment for rectal cancer, with an incidence of 2.6% to 30%. LR usually appears within 2 to 3 years: in particular, 60% to 80% of recurrences occur within the first year and 90% to 93% within 2 years.^[4–6]^ Careful pathological studies have clearly demonstrated that the major cause of LR is the persistence of tumor foci within the mesorectum.^[7,8]^ LR of rectal cancer may be associated with pelvic pain, foul-smelling discharge, tenesmus, and incontinence, and if left untreated it can lead to a very painful death.^[9]^

### 1.2 Clinical pathway

Nowadays, there are different imaging modalities that have been used in the follow-up of rectal cancer, including computed tomography (CT), magnetic resonance imaging (MRI), positron-emission tomography (PET), and positron emission tomography-computed tomography (PET-CT). CT is the diagnostic imaging modality routinely used in follow-up of rectal cancer after surgery, with sensitivity for diagnosing pelvic recurrence around 80% and specificity ranging from 50% to 97%. MRI provides superior soft tissue contrast compared to CT, thus facilitating the distinction of presacral scarring from recurrent tumor. Several studies have investigated the use of fluorodeoxyglucose positron-emission tomography (FDG-PET) for the detection of LR in rectal cancer, with accuracy ranging from 74% to 96%.^[10]^ FDG-PET/CT was shown to help differentiate benign from malignant presacral lesions with a sensitivity of 100% and a specificity of 96%.^[11]^ PET/CT has been demonstrated to improve sensitivity and specificity in the diagnosis of LR,^[10,11]^ but soft tissue contrast is inferior to PET/MRI. In a study by Plodeck et al^[12]^ demonstrates that PET/MRI, a new imaging modalities, shows promising accuracy in the diagnosis of LR of rectal cancer, with a sensitivity and specificity of 94% in this setting.

### 1.3 Why perform this review?

According to the results demonstrated by several studies, the diagnosis of LR can be challenging. The main reason may be based on the fact that some patients develop anastomotic leaks or chronic fistulas, rendering the diagnosis of LR difficult because of extensive post-inflammatory/therapeutic changes and scarring in the pelvic region.^[11,13–16]^ Moreover, the study conducted by Kahi et al^[17]^ demonstrated that it is unclear which modality is better, or what the ideal surveillance intervals should be, although EUS has the potential for detection of extraluminal recurrence before the development of intraluminal endoscopic findings. Some studies also report that approximately 10% of rectal cancer recurrences are diagnosed by EUS only, and missed by other modalities, including proctoscopy.^[18,19]^

A meta-analysis of diagnostic tests represents a powerful tool to summarize findings in the literature by taking into account and enabling analysis of differences between studies. This review may help clinicians identify the most suitable imaging modalities for the diagnosis of patients with LR of rectal cancer.

### 1.4 Objectives

The objective of this meta-analysis is to assess the value of different imaging modalities, including CT, MRI, PET, and PET-CT in the diagnosis and management of patients with LR of rectal cancer.

## 2 Methods/design

We will perform a comprehensive literature search for relevant studies and then screen and select studies for inclusion against eligibility criteria. Data extraction will be performed in duplicate on the selected studies with meta-analysis and report writing. We will adhere to standards of the Preferred Reporting Items for Systematic Reviews and Meta-Analyses (PRISMA) in reporting the findings of this review.^[20]^ The content of this protocol follows the Preferred Reporting Items for Systematic Review and Meta-Analysis Protocols (PRISMA-P) recommendations.^[21]^ This review is registered with the International Prospective Register of Systematic Reviews (PROSPERO).^[22]^ The registration number is CRD42018104918.

### 2.1 Inclusion criteria

#### 2.1.1 Types of studies

We will include reports of cross-sectional studies or case-controlled studies reporting the diagnostic accuracy of ≥1 imaging methods for the diagnosis of LR from rectal cancer, with biopsy and/or follow-up as the reference standard. Reports of studies in which sensitivity and specificity were reported will be included in the review but excluded from the meta-analysis of sensitivity and specificity estimates.

#### 2.1.2 Participants

We will include reports of studies of patients with histological-proven rectal cancer with a suspected LR.

#### 2.1.3 Index tests

We will include only studies of CT, MRI, PET, or PET-CT for the evaluation of the LR of rectal cancer.

#### 2.1.4 Target conditions

LR of rectal cancer is the target condition.

#### 2.1.5 Reference standards

The reference standard used to confirm the presence of the target condition in this study is biopsy and/or follow-up.

#### 2.1.6 Exclusion criterion

(1) Repeated publication.
(2) The control group used a non-criterion standard diagnostic test.
(3) Unable to extract relevant data.
(4) Studies are reviews or abstracts.
(5) Studies published before 1990.

### 2.2 Data sources and search strategy

The Cochrane Library, PubMed, EMBASE, and Chinese Biomedical Literature Database (CBM) will be searched from their inceptions to April 2018. Two reviewers will develop the basic search strategy and full details of the search strategy regarding Cochrane Library, PubMed, and EMBASE will be displayed in Supplementary 1. Additionally, we will handsearch reference lists of included articles and relevant review articles identified through the search and the “related articles” function in PubMed. There will be no language restrictions on our search.

### 2.3 Data collection and analysis

#### 2.3.1 Selection of studies

Literature search records will be imported into ENDNOTE X7 literature management software. Two review authors will independently screen the titles and abstracts of retrieved publications to identify potentially eligible studies for inclusion. The same 2 reviewers will retrieve full-text reports of potentially eligible studies and independently determine study inclusion or exclusion. Any disagreement will be resolved by a third reviewer (J-HT).

#### 2.3.2 Data extraction and management

We will extract the number of true-positives, true-negatives, false-positives, and false-negatives for each index test evaluated in each study to construct 2 × 2 tables using Microsoft Excel 2013 (Microsoft Corp, Redmond, WA, www.microsoft.com). If such data were not provided by the trial authors, we will calculate the number of true-positives, true-negatives, false-positives, and false-negatives from the summary estimates of sensitivity and specificity of the index test, if available. For studies in which only a subgroup of patients were included in the review, we will extract, analyze, and present data for this subgroup only.

Two authors will independently extract the data used for study quality assessment and statistical analysis (data from 2 × 2 tables) and resolve discrepancies by discussion or consultation of third reviewers until a consensus reached. We will extract data, using a predesigned form, including general information about the study including the first author's name, publication year, and financial support of articles etc; the patient characteristics such as mean age, sex, basic treatments; the details of the index test and reference test including its characteristic and whether provided blinding and the process of the diagnostic methods, among others.

#### 2.3.3 Assessment of methodological quality

QUADAS-2 will be used for the methodological quality assessment of included studies.^[23]^ The same 2 review authors will independently collect the information needed to assess the methodological quality of each study using signaling questions (yes/no/unclear) with predefined rules. We will resolve disagreements on the signaling questions by discussion with a third author until a consensus was reached. In keeping with Cochrane DTA Working Group recommendations, no summary score was calculated because this obscures the importance of individual quality and can lead to inaccurate conclusion.^[24,25]^ Thus, we will just summarize the methodological quality assessment of each included study.

### 2.4 Statistical analysis and data synthesis

We will enter data for the 2 x 2 tables into Stata/SE version 12.0 and plot estimates of sensitivity, specificity, positive likelihood ratio (PLR), negative likelihood ratio (NLR), and diagnostic odds ratio (DOR) of CT, MRI, PET, and PET-CT, as well as different sequences of MRI on forest plots and in the summary receiver-operating characteristic(ROC) space to represent the variability in diagnostic test accuracy within and between studies. We will fit the hierarchical bivariate model described by Reitsma et al, 2005^[26]^ by use of Stata/SE version 12 (using the user-written program “metandi”), which allowed for calculating summary estimates of sensitivity and specificity and the associated 95% confidence intervals (CIs). We will also report the estimate of the correlation between sensitivity and specificity. We will put the results from the bivariate model into RevMan 5.3 to provide plots of the estimated summary points and confidence regions, superimpose on the study-specific estimates of sensitivity and specificity in the ROC space, and use the hierarchical summary ROC model to produce summary estimates of sensitivity and specificity. Then we will conduct an indirect comparison to assess the value of different imaging modalities in the diagnosis of LR in terms of sensitivity, sensitivity, PLR, NLR, DOR, and AUC.

We will include the same study in the same meta-analysis more than once if one study reported different index tests. We will present results in groups according to commercial test name. At the same time, sample size funnel plots and associated regression tests for asymmetry will be conducted to detect publication bias.^[27]^

## 3 Discussion

At present, it is not clear whether imaging is beneficial during the surveillance of patients after rectal cancer surgery and trials to establish the role of imaging are ongoing.^[10,28–30]^ Noninvasive imaging modalities such as pelvic CT, MRI, PET, and PET-CT have been proved to be important and widely used as diagnostic tools in the assessment of LR of rectal cancer. However, what is the diagnostic accuracy of CT, MRI, PET, and PET-CT for patients with LR of rectal cancer and which imaging modality is the best choice for patients with such conditions?

By answering these questions, we will conduct a meta-analysis of diagnostic tests with a comprehensive literature search and an indirect comparison between different imaging modalities of CT, MRI, PET, and PET-CT, as well as different sequences of MRI. We hope to provide effective information for clinicians to figure out the diagnostic accuracy of imaging methods above and to recommend the optimal approach for the surveillance in the patients with rectal cancer.

## Acknowledgments

The authors express their thanks to the Evidence-based Medicine Center of Lanzhou University for the assistance with the design of the literature searches.

## Author contributions

HS, XM and JT conceived the idea for this study; BW, JW, JT and HS designed the meta-analysis; XM, JL, YG and JT provided statistical advice and input; HS and XM drafted the protocol; JT and YG reviewed the protocol and provided critical feedback.

## Supplementary Material

Supplemental Digital Content
