# Supplementary material for: Comparison of the diagnostic efficiency for local recurrence of rectal cancer using CT, MRI, PET and PET-CT: A systematic review protocol
Source: Medicine (Baltimore). 2018 Nov 30;97(48):e12900. doi: 10.1097/MD.0000000000012900 (PMC6283203; doi:10.1097/MD.0000000000012900)
Supplement: Supplemental Digital Content [file medi-97-e12900-s001.docx]

**Comparison of the diagnostic efficiency for local recurrence of rectal cancer using CT, MRI, PET and PET-CT：A systematic review protocol**

Hongsheng Shao, MD^a^, Xueni Ma, MD^b^, Ya Gao, MM^c^, Jiancheng Wang, MD^d^, Jiarui Wu, MD^e^, Bo Wang, MD^f^, Jipin Li, MD^b^, and Jinhui Tian, PhD^c^ *

**Supplementary 1: search strategy regarding PubMed**

#1 "Rectal Neoplasms"[Mesh]

#2"rect* neoplasm*"[Title/Abstract] OR "rect* canc*"[Title/Abstract] OR "rect* carcinom*"[Title/Abstract] OR "rect* adenocarc*"[Title/Abstract] OR "rect* tumor*"[Title/Abstract] OR "rect* tumour*"[Title/Abstract] OR "rect* sarcom*"[Title/Abstract]

#3 #1 OR #2

#4 "Tomography, X-Ray Computed"[Mesh]

#5 "computed tomography"[Title/Abstract] OR "computed tomograph*"[Title/Abstract] OR "comput* tomography"[Title/Abstract] OR "comput* tomograph*"[Title/Abstract] OR "computer assisted tomography"[Title/Abstract] OR "comput* assisted tomography"[Title/Abstract] OR CT[Title/Abstract] OR CAT[Title/Abstract] OR "computer assisted tomograph*"[Title/Abstract] OR "comput* assisted tomograph*"[Title/Abstract]

#6 #4 OR #5

#7 "Magnetic Resonance Imaging"[Mesh]

#8 nuclear magnetic resonance imaging[Title/Abstract] OR NMRI[Title/Abstract] OR NMR imaging[Title/Abstract] OR magnetic resonance imaging[Title/Abstract] OR MR tomography[Title/Abstract] OR MRI scans[Title/Abstract] OR MRI scan[Title/Abstract] OR MRI[Title/Abstract] OR functional MRI[Title/Abstract] OR functional MRIs[Title/Abstract] OR chemical shift imaging[Title/Abstract] OR magnetization transfer contrast imaging[Title/Abstract]

#9 #7 OR #8

#10 "Positron-Emission Tomography"[Mesh]

#11 pet*[Title/Abstract] OR petscan*[Title/Abstract] OR (Positron*[Title/Abstract] AND emission*[Title/Abstract]) OR (Positron*[Title/Abstract] AND tomography*[Title/Abstract]) OR (tomograph*[Title/Abstract] AND emission*[Title/Abstract])

#12 #10 OR #11

#13 "Sensitivity AND Specificity"[Mesh] OR "False Positive Reactions"[Mesh] OR "False Negative Reactions"[Mesh] OR "ROC Curve"[Mesh] OR "Predictive Value of Tests"[Mesh]

#14 sensitivity[Title/Abstract] OR specificity[Title/Abstract] OR receiver operating characteristic[Title/Abstract] OR receiver operator characteristic[Title/Abstract] OR predictive value*[Title/Abstract] OR roc[Title/Abstract] OR pre-test odds[Title/Abstract] OR pretest odds[Title/Abstract] OR pre-test probability*[Title/Abstract] OR pretest probability*[Title/Abstract] OR post-test odds[Title/Abstract] OR posttest odds[Title/Abstract] OR post test probabilit*[Title/Abstract] OR posttest probabilit* [Title/Abstract] OR likelihood ratio*[Title/Abstract] OR positive predictive value*[Title/Abstract] OR negative predictive value*[Title/Abstract] OR false negative*[Title/Abstract] OR false positive*[Title Abstract] OR true negative*[Title/Abstract] OR true positive*[Title/Abstract] OR fn[Title/Abstract] OR fp[Title/Abstract] OR tn[Title/Abstract] OR tp[Title/Abstract]

#15 #13 OR #14

#16 #6 OR #9 OR #12

#17 #3 AND #15 AND #16
